# Do cleft lip and palate patients opt for secondary corrective surgery of upper lip and nose, frequently?

**DOI:** 10.1186/1746-160X-9-38

**Published:** 2013-12-09

**Authors:** Emeka Nkenke, Florian Stelzle, Elefterios Vairaktaris, Christian Knipfer

**Affiliations:** 1 Department of Oral and Maxillofacial Surgery, Erlangen University Hospital, Glueckstr. 11, 91054 Erlangen, Germany; 2 Department of Oral and Maxillofacial Surgery, University of Athens Medical School, Attikon Hospital, Athens, Greece

**Keywords:** Cleft lip and palate, Upper lip, Facial aesthetics, Nose, Secondary corrective surgery

## Abstract

**Purpose:**

This prospective study was aimed at assessing cleft lip and palate (CLP) patients’ opinions and attitudes towards their upper lip and nose and the number of secondary corrective surgical interventions electively undertaken to upper lip and nose that were carried out during a 2 year follow-up period.

**Materials and methods:**

During a 2 year follow-up period CLP outpatients were recruited for the study who attended follow-up examinations at a cleft lip and palate craniofacial center and received a recommendation for secondary corrective facial surgery. The participants filled in a questionnaire that included questions regarding the patients’ opinions and attitudes towards appearance of lip and nose and need for secondary corrective facial surgery. During an additional interval of 2 years the rate of patients who underwent secondary corrective surgery to lip and nose was documented.

**Results:**

Out of 362 CLP patients 37 (mean age 13.6 ± 7.6 years) received a recommendation for secondary corrective surgery to upper lip and/or nose. 22 patients (mean age 12.6 ± 6.3 years) filled in the questionnaire (response rate of 62.1%). The satisfaction with the overall facial appearance following the first corrective operation was statistically significantly better than the satisfaction with the nose (p = .016). The satisfaction with facial symmetry (5.6 ± 2.0) did not differ statistically significantly from the overall satisfaction with the facial appearance (6.2 ± 1.8; p = .093). Significantly fewer patients (n = 9) opted for corrective surgery compared to the number of patients who got the recommendation to have secondary corrective surgery done (n = 22, p < .0005).

**Conclusions:**

The findings of the present study may reflect a high overall patient satisfaction with the primary treatment outcome following surgery for CLP. Perceived patient need for secondary operation for the lip/nose may be as low as 5%.

## Introduction

Facial aesthetics is a relevant aspect in a person’s general perception of life [1]. There is a growing popularity of cosmetic surgery procedures all around the world. Individual motivations to opt for aesthetic plastic surgery procedures include the desire to increase self-confidence, self-esteem, and social interactions [2]. One of the major goals of treatment of patients with cleft lip and palate malformations aims at a comparable aspect: the achievement of an unobtrusive facial appearance. By adopting secondary corrective facial surgery efforts are made to achieve psychological and social well-being for the patient as well as his or her family [3]. The different treatment concepts that are followed during childhood, adolescence, and adulthood finally converge in the aim of establishing an unobtrusive facial appearance [4].

A number of different studies have evaluated the facial appearance and/or satisfaction of treated cleft lip and palate patients [5-14]. The results are conflicting. Some authors report that there are no significant differences between aesthetic ratings of professionals and lay persons [6]. On the other hand, it has been shown that professionals rate treated cleft lip and palate patients significantly less attractive than lay persons [5]. A third kind of study reveals that cleft lip and palate patients are less satisfied with their facial appearance than professionals are [8]. From the conflicting data in the current literature it has been concluded that a better understanding of the differences in facial aesthetics perceptions would be a relevant aid in revisional cleft treatment planning as far as facial aesthetics are concerned. So far, information is missing on the number of patients suffering from cleft lip and palate malformations who finally have secondary corrective facial surgery done. The present prospective study aimed at assessing.

i) cleft lip and palate patients’ opinions on and their attitude towards their facial appearance, and

ii) the number of secondary corrective surgical interventions to upper lip and/or nose that were carried out during a 2 year follow-up period.

## Material and methods

The study was approved by the institutional ethics committee of the University of Erlangen-Nuremberg, Germany. It included patients with repaired cleft lip and palate malformations who were treated at the cleft lip and palate craniofacial center of the Erlangen University Hospital, Germany. Participants were recruited from all consecutive patients who joined a follow-up examination between January 2009 and December 2010.

The follow-up examinations were carried out by an interdisciplinary team of an oral and maxillofacial surgeon, an oto-rhino-laryngologist and an orthodontist. A checklist that included the items “performed surgical interventions” and “indication for secondary corrective facial surgery” was used to perform the examinations in a standardized fashion. Every patient was asked if he or she felt the need for secondary corrective facial surgery and wanted to get medical advice concerning this aspect. If the answer was “yes”, the Asher-McDade esthetic index was used to score nasolabial appearance [15]. In this index, 4 components of the nasolabial area are scored, separately, on frontal and lateral view photographs (nasal form, frontal view; deviation of the nose, frontal view; shape of the vermillion border and contour of the upper lip, frontal view; nasal profile including upper lip, lateral view) Each feature was rated on a 5-point scale (1, very good appearance; 2, good appearance; 3, fair appearance; 4, poor appearance; 5, very poor appearance). If a single feature was rated ″4″ or ″5″, secondary corrective surgery was recommended. For further statistical analysis only the rating that led to the recommendation of secondary corrective surgery was chosen.

Possibilities and limitations of the prospective operation were explained to each patient in the light of the individual case. Patients who took secondary corrective surgery to lip and/or nose into consideration were eligible for further participation in the study. Each participant had to sign an informed consent form. The demographic data of the included patients were compiled (Tables 1, 2, 3, 4 and 5).

**Table 1:** Demographic data of the 37 patients who were eligible for participation in the study

		**Patient cohort that received a recommendation for secondary corrective surgery**	**Patient cohort that returned questionnaires**	***p***
Gender distribution	Female	17 (46%)	11 (50%)	
	Male	20 (54%)	11 (50%)	.763
	Total	37 (100%)	22 (100%)	
Age (years) Mean (SD)	Female	12.1 (5.7)	11.2 (5.3)	.906
	Male	15.0 (8.8)	14.1 (7.1)	.867
	Total	13.6 (7.6)	12.6 (6.3)	.882
No. of patients who underwent secondary corrective surgery	Female	8 (50%)	5 (56%)	
	Male	8 (50%)	4 (44%)	.790
	Total	16	9	

SD, standard deviation.

**Table 2:** Distribution of clefts in the cohorts of patients that received a recommendation for secondary corrective facial surgery and that returned the questionnaire

	**Patient cohort that received a recommendation for secondary corrective facial surgery**		**Patient cohort that returned questionnaires**		***p***
	**Unilateral cleft lip and palate**	**Bilateral cleft lip and palate**	**Unilateral cleft lip and palate**	**Bilateral cleft lip and palate**	
Female	11	6	9	2	.328
Male	12	8	8	3	.479
Total	23	14	17	5	.230

**Table 3:** Comparison of demographic data between patients who returned and who did not return the questionnaire

		**Patient cohort that returned questionnaires**	**Patient cohort that did not return questionnaires**	***p***
Age (years) Mean (SD)		15.1 (9.2)	12.6 (6.3)	.414
Gender	Female	11	6	.676
	Male	11	8	
Kind of cleft malformation	Unilateral	17	6	.220
	Bilateral	5	9	
Feature recommended for revision	Nose	12	7	.890
	Lip	15	8	

**Table 4:** Demographic data of patients with unilateral and bilateral cleft lip and palate malformations who returned the questionnaire

	**Unilateral CLP**		**Bilateral CLP**	
Gender	N	Age (years) mean (SD)	N	Age (years) mean (SD)
Female	9	11.0 (5.3)	2	12.0 (7.0)
Male	8	13.75 (8.0)	3	15.0 (5.0)
Total	17	12.3 (6.7)	5	13.8 (5.2)

The difference in mean age between the 17 patients with unilateral cleft lip and palate malformations and the 5 patients with bilateral cleft lip and palate malformations is not statistically significant (p = .651).

**Table 5:** Demographic data of patients who returned the questionnaire distinguishing patients who underwent and who did not undergo secondary corrective facial surgery

	**Corrective surgery**		**No corrective surgery**	
Gender	N	Age (years) mean (SD)	N	Age (years) mean (SD)
female	5	15.80 (1.1)	6	7.33 (4.1)
male	4	20.5 (5.9)	7	10.4 (4.9)
total	9	17.9 (4.4)	13	9.0 (4.6)

The difference in mean age between the 9 patients who underwent corrective surgery and the 13 patients who did not is statistically significant (p < .0005).

A questionnaire was distributed by surface mail to the patients where the indication for secondary corrective facial surgery was seen. The questionnaire was designed to assess the patients’ opinions on and the attitude towards their facial appearance (Table 3). Patients and their parents were informed in a personalized cover letter that participation in the study was voluntary and that individual responses would be confidential. A stamped self-addressed return envelope was included. No personal incentive was offered. Patient and/or parent informed consent and participant assent were obtained.

The questionnaire covered the interdisciplinary team’s recommendation for secondary corrective surgery upper lip and/or nose, the patients’ satisfaction with the appearance of the face, nose and upper lip, and their desire for secondary corrective surgery. 6 items were included that had to be answered either by checking the provided answers or on a 9-point rating scale. The questionnaire was based on that used by Meyer-Marcotty et al. [16]. When the questionnaire was not returned within 30 days a follow-up letter was sent out. When the questionnaire was not returned after additional 30 days, it was assumed that the respective patients were not willing to participate in the study.

The response rate to the questionnaire was calculated. The number of patients who were operated on for secondary corrective facial surgery within 24 months after the indication had been established or who were willing to be operated on, and the kind of operation were documented.

### Statistical analysis

Mean values were given with standard deviations. For comparison of continuous variables in paired samples, the Wilcoxon test was used, while for unpaired samples the Mann–Whitney-U test was adopted. The χ^2^ test was used to test if there was a statistically significant difference in gender distribution, kind of clefts, and decision for or against secondary corrective surgery in the different groups. In order to assess correlations the Pearson correlation coefficient was calculated. P-values less than or equal to .05 were considered significant. Cronbach’s α analysis was performed to assess reliability of the questionnaire. α-values of .7 or higher are in the acceptable range recommended by the literature [17]. α-values above .8 reflect a high reliability. All calculations were made using IBM SPSS statistics 20 (IBM, Armonk, NY, U.S.A.).

## Results

A total of 362 patients attended a follow-up examination at the cleft lip and palate craniofacial center between January 2009 and December 2010. In 37 patients at least 1 feature of the Asher-McDade esthetic index was rated ″4″ or ″5″. To these patients secondary corrective surgery was recommended (17 female, 20 male, mean age 13.6 ± 7.6 years, Tables 1 and 2). The recommendations comprised rhinoplasty in 19 cases and lip revision in 23 cases. To each of the 37 patients a questionnaire was sent out by surface mail. 12 questionnaires were returned within the first 30 days. After that time, the non-responders received a follow-up letter. Additional 11 questionnaires were returned within the next 30 days. The final response rate after 2 months was 62.1%. 1 questionnaire had to be excluded from further analysis because personal information of the patient has not been stated by the patient in the response letter. Cronbach’s α of .792 indicated an acceptable reliability of the questionnaire. The 22 returned questionnaires that were suitable for further analysis belonged to 11 female and 11 male patients with an average age of 12.4 ± 6.3 years (Table 1). The mean age of the female and the male patients did not differ statistically significantly (p = .300). In 12 cases secondary corrective surgery to the nose and in 15 cases secondary corrective surgery to the upper lip had been recommended by the interdisciplinary team. The results reveal that the cohort of 37 patients who were eligible for the study, did not differ statistically significantly from the patients who finally returned the questionnaire as far as age (p = .882), gender (p = .763), and the kind of cleft malformation (p = .230) were concerned (Tables 1, 2 and 3). Comparable results were found when the patients who returned the questionnaire and the patients who did not return the questionnaire, were compared (age, p = .414; gender, p = .676; kind of cleft malformation, p = .220). There was no difference in age when the patients with unilateral cleft lip and palate malformations and the patients with bilateral cleft lip and palate malformations were compared who had returned the questionnaire (p = .651, Table 4).

**Figure 1:**
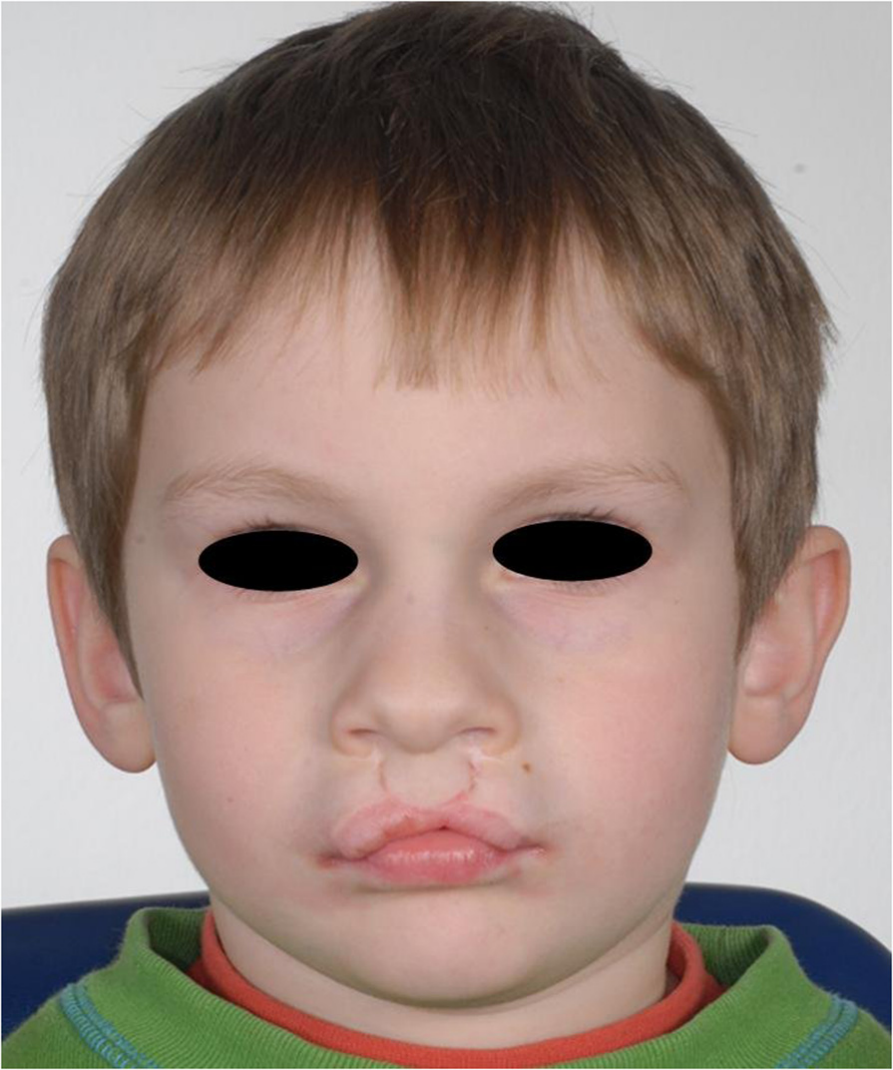
8-year old patient scheduled for corrective surgery of the upper lip.

**Figure 2:**
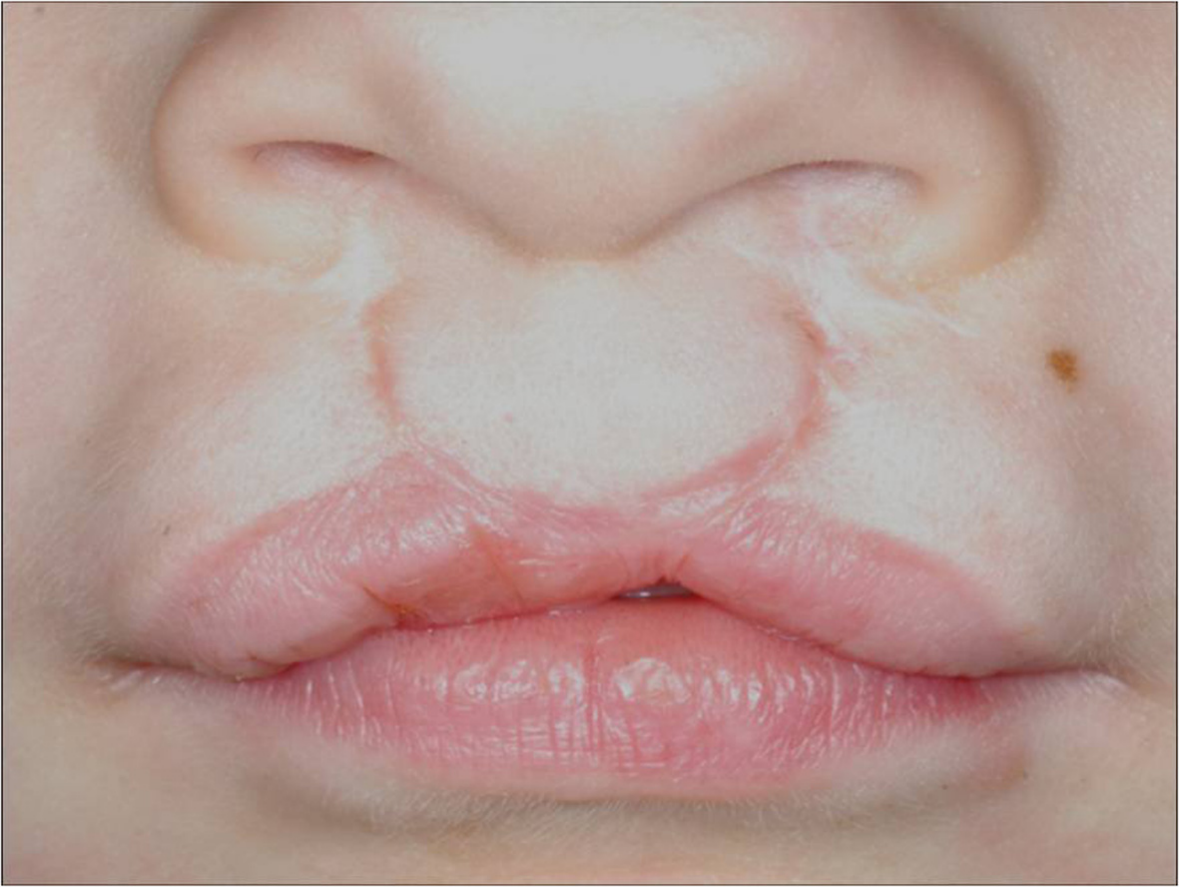
**Detail of the upper lip of the patient in Figure **1**showing a whistling defect.**

**Figure 3:**
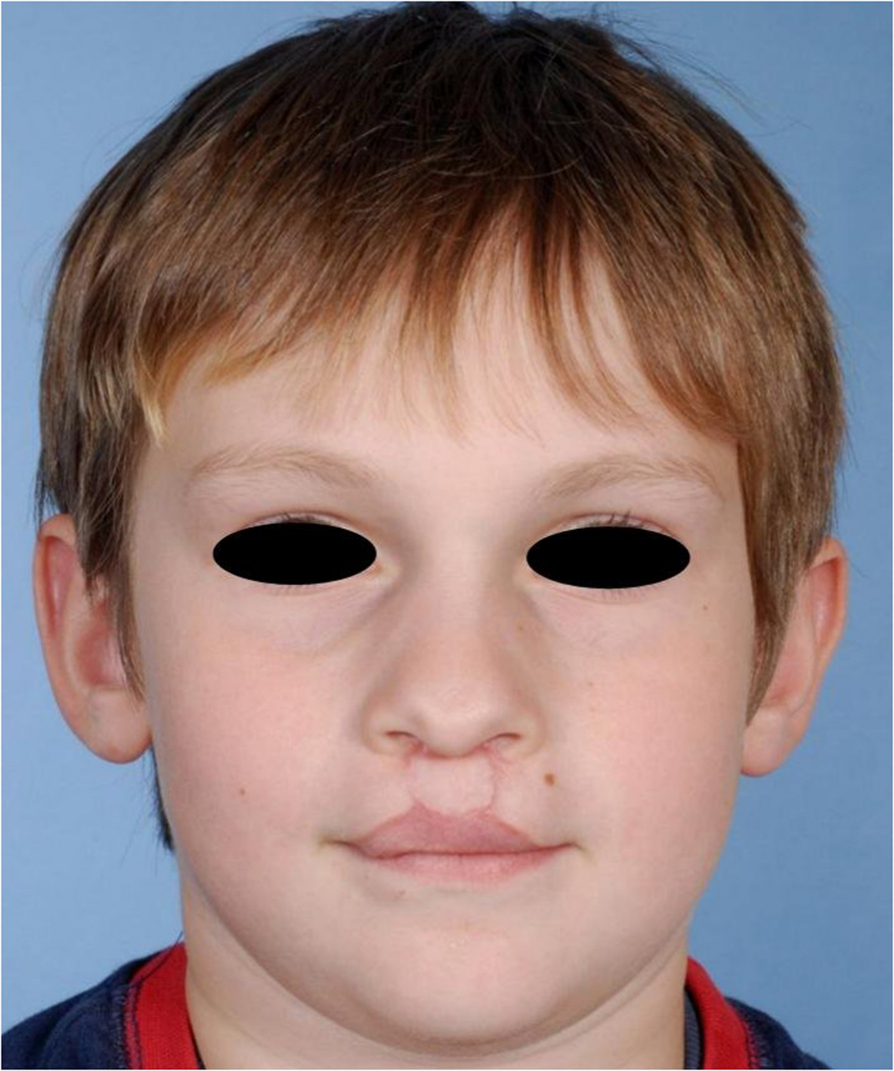
Postoperative situation following corrective surgery of the upper lip.

**Figure 4:**
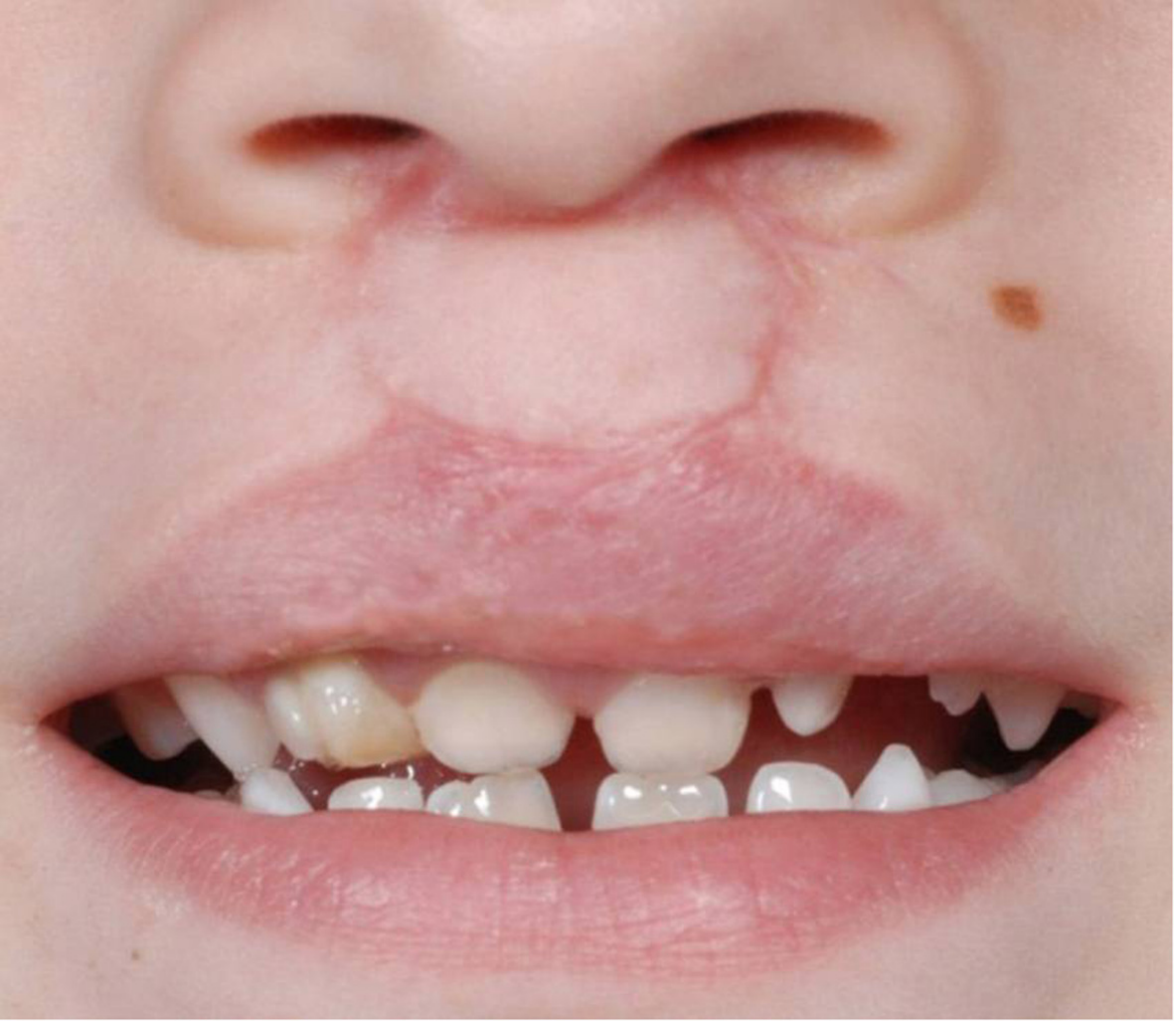
**Detail of the postoperative situation in Figure **3**with complete repair of the whistling defect.**

**Figure 5:**
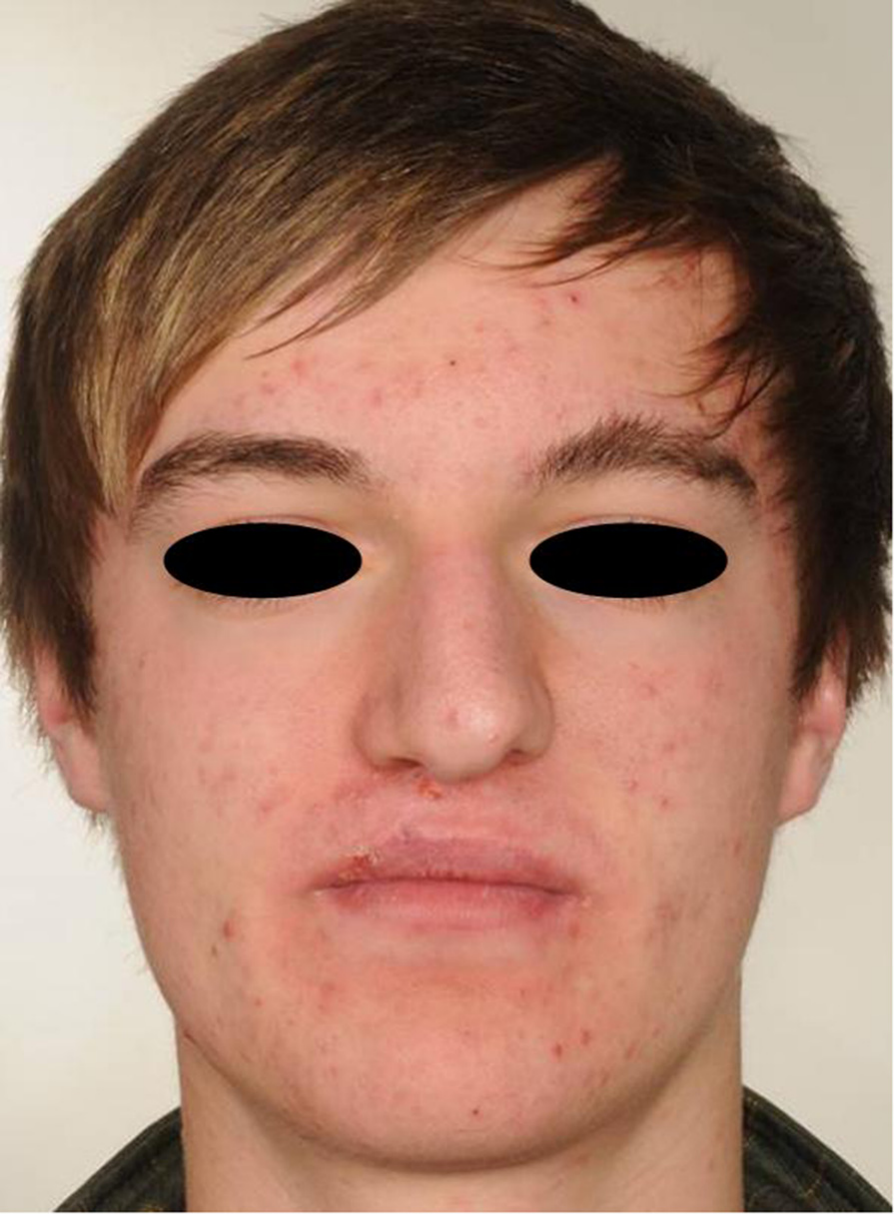
Frontal view of a 15-year old patient scheduled for rhinoplasty.

**Figure 6:**
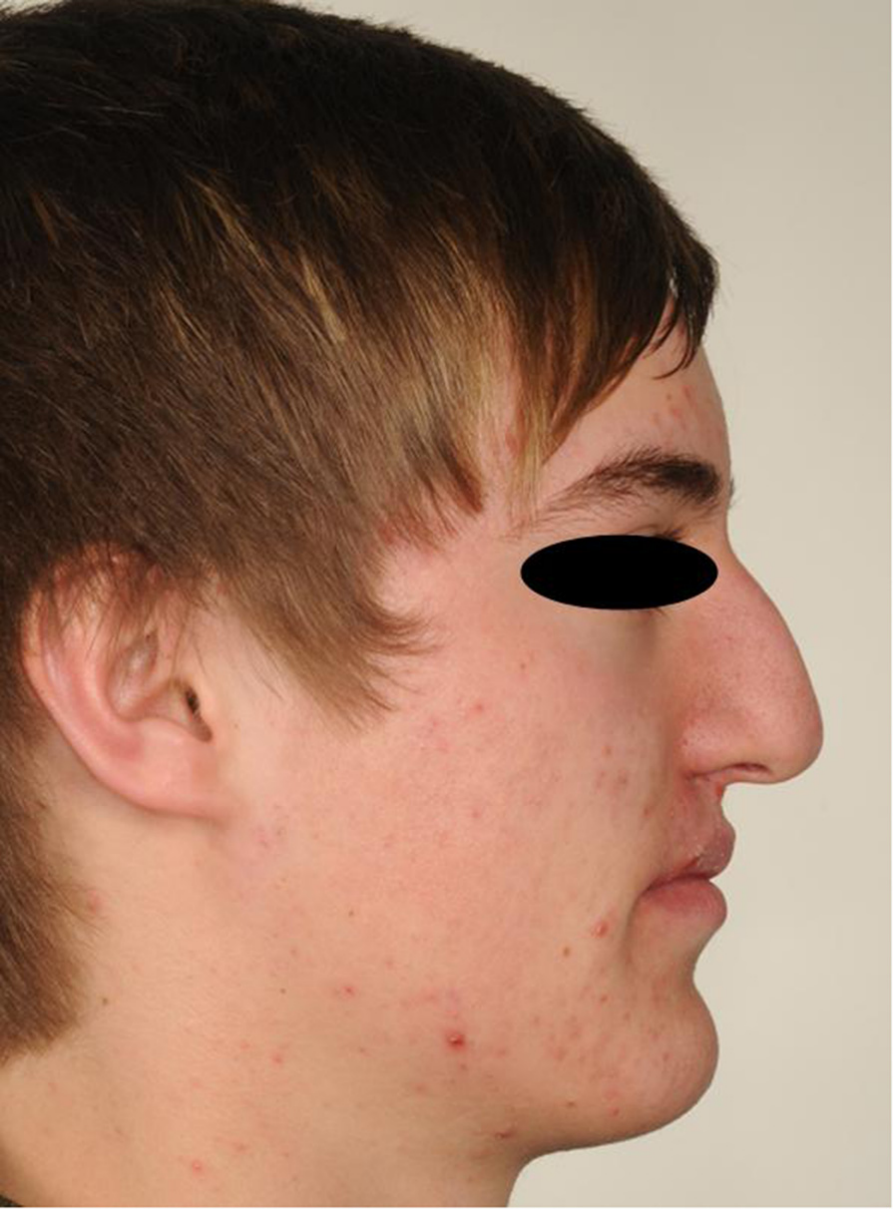
Lateral view of the patient.

**Figure 7:**
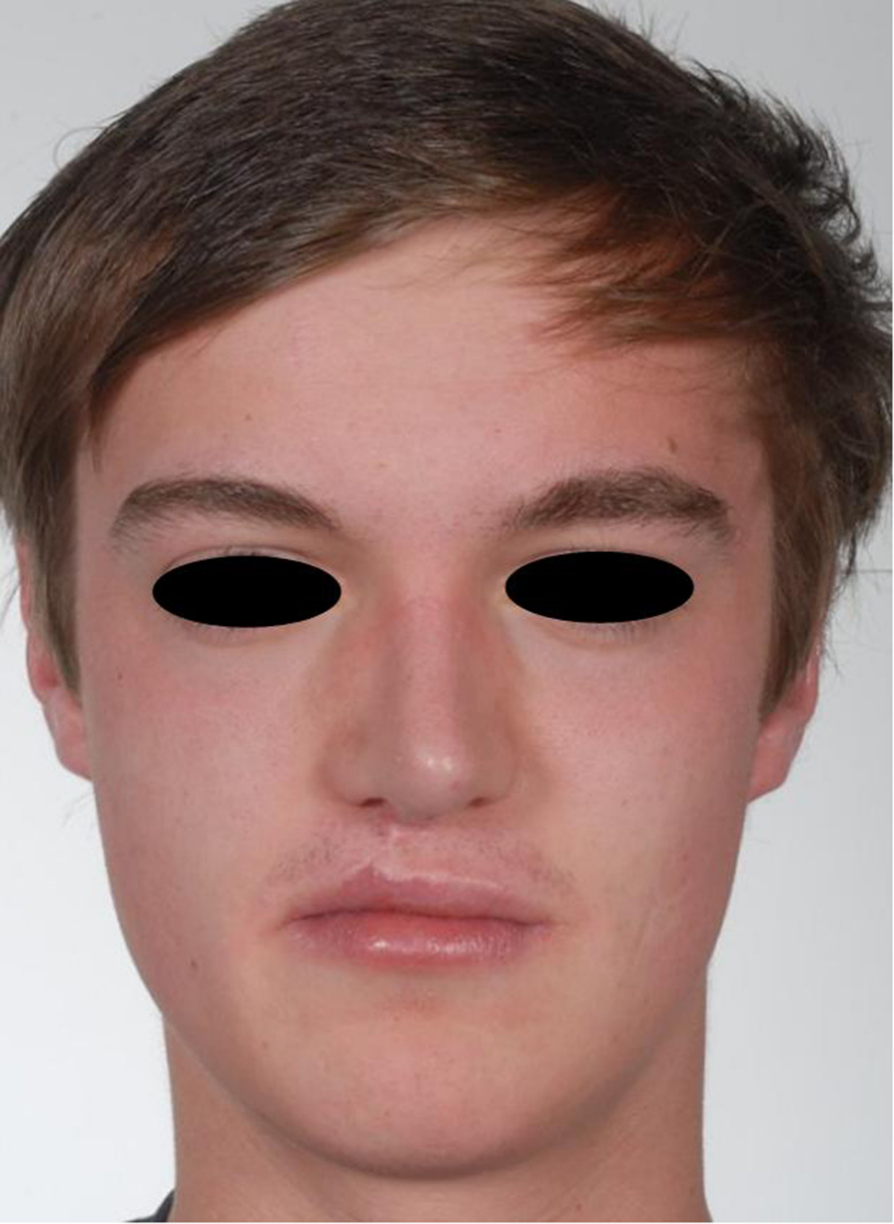
Postoperative frontal view of the patient following rhinoplasty.

**Figure 8:**
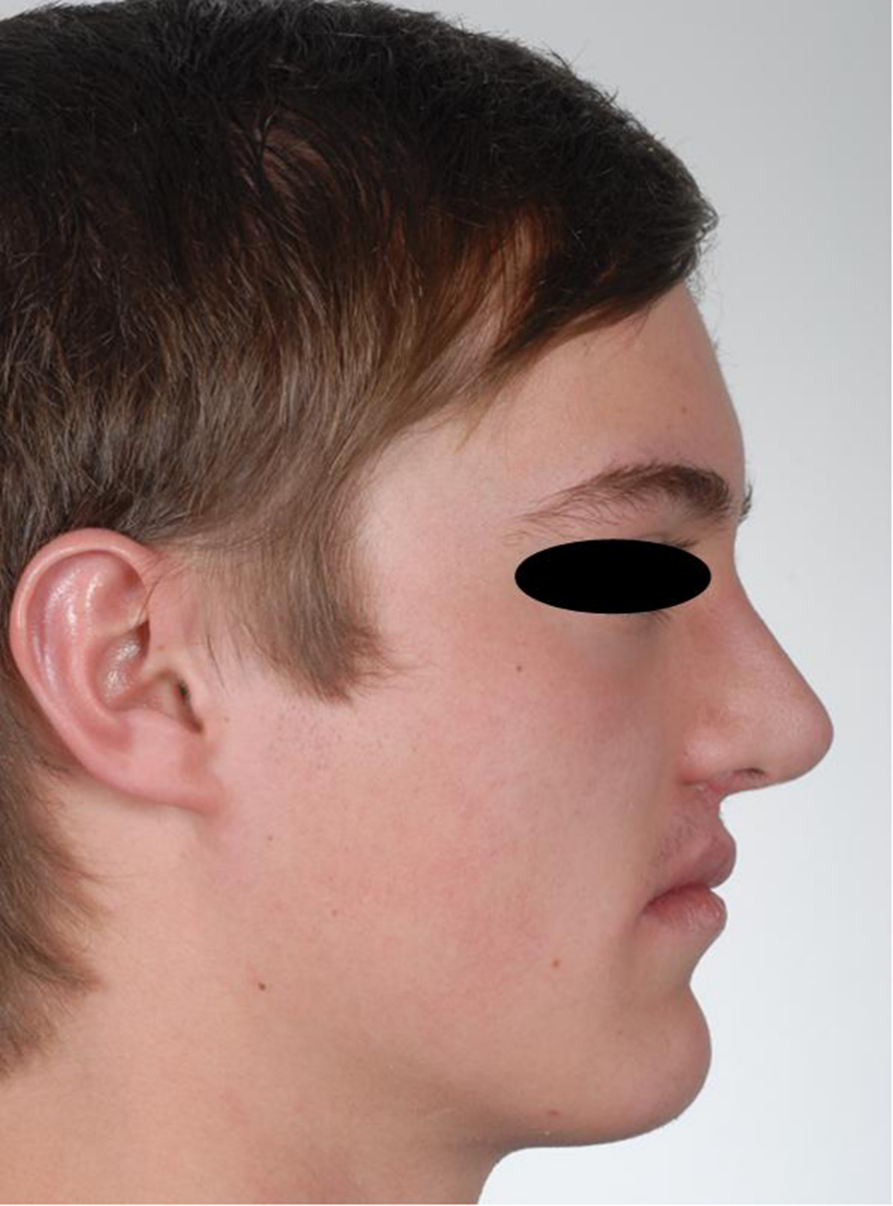
Postoperative profile of the patient.

In the cohort of the responders the number of recommendations did not differ statistically significantly between nose and upper lip (p = .234). During the follow-up period 9 of the 22 responders decided to have secondary corrective facial surgery done (Figures 1, 2, 3, 4, 5, 6, 7 and 8, Table 5). These were significantly less patients than the complete cohort that had received a recommendation for secondary corrective facial surgery (p < .0005). In the 22 patients who had received a recommendation for corrective surgery there was no statistically significant correlation between Asher-McDade esthetic index ratings and the decision for or against secondary corrective surgery in this group (p = .085).

10 cases of rhinoplasty (4 unilateral cleft lip and palate patients, 6 bilateral cleft lip and palate patients) and 8 lip revisions (4 unilateral cleft lip and palate patients, 4 bilateral cleft lip and palate patients) were performed. The number of female and male patients who were operated on did statistically not differ on a significant level (p = .886). The patients who underwent corrective surgery were significantly older than the patients who did not undergo corrective surgery during the observation period (p < .0005, Table 5).

The results from the questionnaire showed that only 2 male patients were completely satisfied with their overall facial appearance. 5 male and 6 female patients considered their nose the least satisfying feature of their face, while 4 male and 5 female patients reported their upper lip to be the least satisfying feature (Question 1, Table 6). There was no gender difference for both features (p = .912). All female and 9 male patients stated that they considered a recommended further improvement of their facial appearance congruent to their personal needs (Question 2; Table 5). 15 patients tended to choose an improvement of the nose, while 5 patients tended to opt for an improvement of the upper lip (p = .007). There was no gender difference for the preference of the correction of the nose or the lip (p = .339; Question 3, Table 5).

**Table 6:** Results of the Questionnaire given separately for male and female patients

**Question 1**	**Gender**	**N**	**Nose**	**Upper lip**	**None, completely satisfied**	***p***
Which part of your face do you consider the least satisfying?	Female	11	6	5	/	.912
	Male	11	5	4	2	
Question 2	Gender	N	yes	no	Question not answered	*p*
Does the recommendation for corrective facial surgery meet your personal needs?	Female	11	11	/	/	.220
	Male	11	9	1	1	
Question 3	Gender	N	Nose	Upper lip	Question not answered	p
If you think about corrective facial surgery, which feature of your face should be improved (nose/upper lip)?	Female	11	7	3	1	.339
	Male	11	8	2	1	
Question 4	Gender	N	Mean (SD)			*p*
4.1 How satisfied are you with your facial aesthetics?	Female	11	6.6 ± .9			0.311
	Male	11	5.8 ± 2.4			
	Total	22	6.2 ± 1.8			
4.2 How symmetrical do you consider your face?	Female	11	5.9 ± 2.2			0.367
	Male	11	5.3 ± 1.9			
	Total	22	5.6 ± 2.0			
4.3 How satisfied are you with the appearance of your nose?	Female	11	5.3 ± 2.9			0.871
	Male	11	5.5 ± 2.2			
	Total	22	5.4 ± 2.5			
4.4 How satisfied are you with the appearance of your upper lip?	Female	11	5.3 ± 2.4			0.752
	Male	11	5.6 ± 1.5			
	Total	22	5.4 ± 1.9			
Question 5	Gender	N	yes		no	*p*
Do you plan to undergo secondary corrective facial surgery within the following 2 years?	Female	11	8		3	.766
	Male	11	9		2	
Question 6	Gender	N	I am satisfied with facial appearance	I am tired of being operated on	I feel too young	*p*
If you do not plan to undergo secondary corrective facial surgery, what is the reason?	Female	11	/	/	/	.091
	Male	11	1	/	3	

For Question 4 answers could be given on a 9-point rating scale (1, maximum satisfaction and symmetry, resp.; 9, minimum satisfaction and symmetry, resp.).

The results of the patients’ self-report for satisfaction with overall facial appearance, facial symmetry nose and lip can be found in Table 6 (Question 4). There were no statistically significant gender differences. For the complete cohort of 22 patients the satisfaction with the overall facial appearance was statistically significantly better than the satisfaction with the nose (p = .016). The satisfaction with the overall facial aesthetics did not differ significantly to the upper lip (p = .924) as well as to the rating of the facial symmetry (p = .093). The degree of satisfaction did not differ statistically significantly between nose and upper lip (p = .593). Nose and upper lip ratings did not differ significantly to the self-perceived satisfaction with the facial symmetry either (p_nose_ = .436, p_upper lip_ = .547).

17 patients stated that they would chose secondary corrective surgery within the next two years while 5 patients did not want to undergo secondary corrective surgery in the near future. There was no statistically significant difference for this aspect between male and female patients (Table 5, Question 5, p = .766). Out of the 5 patients who did not want to have secondary corrective facial surgery, 1 male patient was satisfied with his facial appearance and 3 patients felt being too young for secondary corrective surgery and chose to postpone it. 1 patient did not indicate a reason for refraining from secondary corrective surgery. None of the patients responded that he or she was tired of being operated on (Question 6; Table 6).

8 out of 17 patients indicated that they would make use of this option in the future, although they did not during the follow-up period (3 female, 5 male, mean age 11.00 ± 4.72 years). These patients intended to have corrections made of the nose (5 cases) and the upper lip (6 cases).

When the data of the questionnaire were compared for patients with unilateral and patients with bilateral cleft lip and palate malformations, there was a significant difference as far as the preference of the correction of the nose or the lip was concerned (p = .030; Question 3, Table 7). In 14 out of 17 cases, the patients with unilateral cleft lip and palate malformations indicated that they would choose corrective surgery of the nose. 3 out of 4 patients with bilateral cleft lip and palate malformation would choose a correction of the upper lip. Although the patients with unilateral cleft lip and palate malformations considered their faces to be symmetrical, their ratings were significantly lower than the ratings of the patients with bilateral cleft lip and palate malformations (p = .039; Question 4.2, Table 7).

**Table 7:** Results of the questionnaire given separately for unilateral and bilateral cleft lip and palate patients

**Question 1**	**Type of cleft**	**N**	**Nose**	**Upper lip**	**None, completely satisfied**	***p***
Which part of your face do you consider the least satisfying?	Unilateral	17	10	5	2	.124
	Bilateral	5	1	4	/	
Question 2	Type of cleft	N	yes	no	Question not answered	*p*
Does the recommendation for corrective facial surgery meet your personal needs?	Unilateral	17	16	1	/	.411
	Bilateral	5	4	/	1	
Question 3	Type of cleft	N	Nose	Upper lip	Question not answered	p
If you think about corrective facial surgery, which feature of your face should be improved (nose/upper lip)?	Unilateral	17	14	2	1	.030
	Bilateral	5	1	3	1	
Question 4	Type of cleft	N	Type of Cleft			*p*
4.1 How satisfied are you with your facial aesthetics?	Unilateral	17	6.18 ±1.8			0.216
	Bilateral	5	6.40 ± 1.9			
	Total	22	6.2 ± 1.9			
4.2 How symmetrical do you consider your face?	Unilateral	17	5.1 ± 1.8			0.039
	Bilateral	5	7.2 ± 1.9			
	Total	22	5.6 ± 2.0			
4.3 How satisfied are you with the appearance of your nose?	Unilateral	17	5.3 ± 2.9			0.154
	Bilateral	5	6.8 ± 2.5			
	Total	22	5.4 ± 2.5			
4.4 How satisfied are you with the appearance of your upper lip?	Unilateral	17	5.1 ± 2.0			0.202
	Bilateral	5	6.4 ± 1.7			
	Total	22	5.4 ± 1.9			
Question 5	Type of cleft	N	yes	no		*p*
Do you plan to undergo secondary corrective facial surgery within the following 2 years?	Unilateral	17	13	4		.869
	Bilateral	5	4	1		
Question 6	Type of cleft	N	I am satisfied with facial appearance	I am tired of being operated on	I feel too young	*p*
If you do not plan to undergo secondary corrective facial surgery, what is the reason?	Unilateral	17	1	/	2	.853
	Bilateral	5	/	/	1	

For Question 4 answers could be given on a 9-point rating scale (1, maximum satisfaction and symmetry, resp.; 9, minimum satisfaction and symmetry, resp.).

When the data of the questionnaire were compared for patients who underwent secondary corrective facial surgery and the patients who did not undergo secondary corrective surgery during the observation period, no statistically significant differences could be found (Table 8).

**Table 8:** Results of the questionnaire given separately for patients who underwent secondary corrective facial surgery and who did not

**Question 1**	**Corrective surgery**	**N**	**Nose**	**Upper lip**	**None, completely satisfied**	***p***
Which part of your face do you consider the least satisfying?	Yes	9	4	4	1	.901
	No	13	7	5	1	
Question 2	Corrective surgery	N	yes	no	Question not answered	*p*
Does the recommendation for corrective facial surgery meet your personal needs?	Yes	9	8	/	/	.784
	No	13	12	1	1	
Question 3	Corrective surgery	N	Nose	Upper lip	Question not answered	p
If you think about corrective facial surgery, which feature of your face should be improved (nose/upper lip)?	Yes	9	6	2	1	.0.963
	No	13	9	3		1
Question 4	Corrective surgery	N	Type of Cleft			*p*
4.1 How satisfied are you with your facial aesthetics?	Yes	9	5.78 ±2.3			0.519
	No	13	6.69 ± 1.3			
	Total	22	6.2 ± 1.9			
4.2 How symmetrical do you consider your face?	Yes	9	5.22 ± 2.4			0.437
	No	13	5.85 ± 1.7			
	Total	22	5.6 ± 2.0			
4.3 How satisfied are you with the appearance of your nose?	Yes	9	5.67 ± 3.3			0.652
	No	13	5.15 ± 1.9			
	Total	22	5.4 ± 2.5			
4.4 How satisfied are you with the appearance of your upper lip?	Yes	9	5.67 ± 2.3			0.617
	No	13	5.23 ± 1.6			
	Total	22	5.38 ± 1.8			
Question 5	Corrective surgery	N	yes	no		*p*
Do you plan to undergo secondary corrective facial surgery within the following 2 years?	Yes	9	7	2		.962
	No	13	10	3		
Question 6	Corrective surgery	N	I am satisfied with facial appearance	I am tired of being operated on	I feel too young	*p*
If you do not plan to undergo secondary corrective facial surgery, what is the reason?	Yes	9	/	/	1	.530
	No	13	1	/	2	

For Question 4 answers could be given on a 9-point rating scale (1, maximum satisfaction and symmetry, resp.; 9, minimum satisfaction and symmetry, resp.).

## Discussion

The present prospective study aimed at assessing cleft lip and palate patients’ opinions on and the attitude towards their facial appearance, and their tendency to opt for secondary corrective facial surgery. Based on the identification of patients who were eligible for the study clinical data on these persons were collected and a questionnaire was sent out to compile the relevant data. Although a response rate of 23 of 37 invited patients may seem low, it is exactly within the range that can be expected from the current literature. Response rates of approx. 58% to questionnaires received by surface mail have to be expected [18]. The comparison between the patients who were eligible for the study and the patients who finally returned the questionnaire revealed that there were no statistically significant differences between the 2 cohorts. Consequently, it can be assumed that the results were not biased in a pronounced way by the portion of non-responders. It can be assumed that the bias resulting from the non-responders falls within the normal range. With a Cronbach’s α of .792 the questionnaire had an acceptable reliability revealing that the results obtained in this study are relevant [17].

The interdisciplinary team of the cleft palate craniofacial center recommended secondary corrective facial surgery to approximately 10% of the patients who attended a follow-up examination during a 2-years period (37 out of 362 patients). However, only 4% (16 out of 362) of the patients chose this option during the follow-up period of 24 months. This result is surprising because, historically, cleft lip and palate patients have demonstrated a positive correlation between satisfaction with facial appearance and health related quality of life [9]. Cleft lip and palate patients are more concerned with visible defects than with functional problems. However, keeping in mind that patients with repaired cleft lip and palate malformations feel as socially accepted as do peers without such malformations this low number of patients who decided to have corrective surgery is not surprising [19]. In addition, there are studies that show that patients with cleft lip and palate malformations seem to be relatively satisfied with their body image [8]. In this context, symmetry is an important aspect. Symmetrical faces are perceived as being more attractive [20]. Impaired symmetry might cause significant emotional distress due to unhappiness with facial appearance [7]. However, in the present study the satisfaction with facial symmetry did not statistically significantly differ from the overall satisfaction with the facial appearance. It reached an average value over 5.6 ± 2.0 on a 9-point rating scale indicating a tendency towards satisfaction. It seems that the cohort of patients that was analyzed did not identify their facial symmetry as a major problem.

It has been described in the past that patients with cleft lip and palate malformations often consider their nose unsatisfactory [21]. The present study confirms these findings. Patients were significantly more satisfied with their overall facial appearance than they were with their nose. This fact was especially true for patients with unilateral cleft lip and palate malformations. Consequently, in the present study corrections of the nose were the kind of corrective surgery desired and performed most often. However, the number of patients who actually chose to have secondary corrective surgery of the nose done during the follow-up period was low (9 out of 22 responders to the questionnaire). This finding has also been described previously. Although patients with cleft lip and palate malformations often feel the need for a correction, they often do not have secondary corrective facial surgery done [12].

The present study failed to show a correlation between the professional rating of esthetics as a basis for the recommendation for secondary corrective surgery and the actual decision of the patients for or against corrective surgery. This problem has been addressed in the current literature, previously [5]. It has been hypothesized that although secondary corrective facial surgery is recommended by professionals, the low rate of actual decision for surgery is the consequence of a prolonged treatment course of patients suffering from cleft lip and palate malformations. Multiple previous operations make the patients tired of additional interventions [12]. Although the patients in the present study were explicitly asked if they refrained from surgery as a consequence of multiple previous interventions, none of the patients stated that this aspect was an important reason for their decision. Therefore, the aspect of surgical fatigue seemed to have no relevance in the present study.

The mean age of patients seeking corrective surgery in the present study around the age of 13 years at the edge of puberty is not surprising. This aspect seems to correlate with intensive stigma experiences during adolescence [22]. Facial appearance exerts strong impact on social interaction and personal development [23]. Consequently, facial differences are presumed to negatively affect social encounters and to put individuals at risk for psychological difficulties and impaired quality of life [24]. Research findings confirm that individuals with visible differences are likely to experience stigmatizing behaviors such as staring, avoiding, teasing, and manifestations of pity [25]. However, it has been stated that facial differences do not necessarily lead to major psychological maladjustment [26]. This aspect again might be an explanation for the low number of patients who chose to be operated on in the present study.

In the past, there have been conflicting results as far as a gender difference in the need for secondary corrective facial surgery is concerned. There have been authors who described that female patients with cleft lip and palate malformations deemed corrective surgery significantly less necessary than male patients [5]. On the other hand, it has been shown that female cleft lip and palate patients wished to have corrective surgery twice as often as male patients [12]. In the present study an even distribution between male and female patients was found. There was no gender difference as far as the need for secondary corrective facial surgery was concerned.

The present study adds information on the need of cleft lip and palate patients to undergo secondary corrective facial surgery to the current literature. The major limitation of the study is the low demand for secondary corrective facial surgery that led to low case numbers. There is a chance that the study failed to demonstrate statistical significance for some aspects which might have shown this significance with larger case numbers. Consequently, the study is continued in order to increase the number of included patients allowing a final comprehensive statistical analysis.

## Conclusions

The results of the present study reveal that the need for secondary corrective surgery to upper lip and/or nose is low in the described cohort of patients with cleft lip and palate malformations. Significantly fewer patients opt for corrective surgery compared to the number of patients who got the recommendation to have secondary corrective facial surgery done. These findings might reflect the good overall patient satisfaction with the outcome of primary surgical treatment of cleft lip and palate malformations.

### Consent statement

All patients and parents, resp., included in the study were asked to sign an informed consent form in accordance with Helsinki Declaration.

## Abbreviations

CLP: Cleft lip and palate; SD: Standard deviation.

## Competing interests

The authors declare that they have no competing interests.

## Authors’ contributions

EN, FS, EV and CK conceived of the study, participated in its design and coordination, collected the data, wrote and draft the manuscript. CK performed the statistical analysis. All authors read and approved the final manuscript.
